# Turkish Chest X-Ray Report Generation Model Using the Swin Enhanced Yield Transformer (Model-SEY) Framework

**DOI:** 10.3390/diagnostics15141805

**Published:** 2025-07-17

**Authors:** Murat Ucan, Buket Kaya, Mehmet Kaya

**Affiliations:** 1 Department of Computer Technologies, Vocational School of Technical Sciences, Dicle University, Diyarbakir 21200, Turkey; murat.ucan@dicle.edu.tr; 2 Department of Electronics and Automation, Firat University, Elazig 23119, Turkey; bkaya@firat.edu.tr; 3 Department of Computer Engineering, Firat University, Elazig 23119, Turkey

**Keywords:** medical report generation, chest X-ray, Swin transformer, GPT, Turkish

## Abstract

**Background/Objectives**: Extracting meaningful medical information from chest X-ray images and transcribing it into text is a complex task that requires a high level of expertise and directly affects clinical decision-making processes. Automatic reporting systems for this field in Turkish represent an important gap in scientific research, as they have not been sufficiently addressed in the existing literature. **Methods**: A deep learning-based approach called Model-SEY was developed with the aim of automatically generating Turkish medical reports from chest X-ray images. The Swin Transformer structure was used in the encoder part of the model to extract image features, while the text generation process was carried out using the cosmosGPT architecture, which was adapted specifically for the Turkish language. **Results**: With the permission of the ethics committee, a new dataset was created using image–report pairs obtained from Elazıg Fethi Sekin City Hospital and Indiana University Chest X-Ray dataset and experiments were conducted on this new dataset. In the tests conducted within the scope of the study, scores of 0.6412, 0.5335, 0.4395, 0.4395, 0.3716, and 0.2240 were obtained in BLEU-1, BLEU-2, BLEU-3, BLEU-4, and ROUGE word overlap evaluation metrics, respectively. **Conclusions**: Quantitative and qualitative analyses of medical reports autonomously generated by the proposed model have shown that they are meaningful and consistent. The proposed model is one of the first studies in the field of autonomous reporting using deep learning architectures specific to the Turkish language, representing an important step forward in this field. It will also reduce potential human errors during diagnosis by supporting doctors in their decision-making.

## 1. Introduction

Paragraph-level reporting of medical images is a critical and important process for the diagnosis, follow-up, and treatment planning of diseases [1]. In particular, chest X-ray images are a low-cost and rapid acquisition method widely used for the diagnosis of respiratory diseases. However, in traditional methods, chest X-ray images are examined by specialized physicians and medical reports are written. This process is a critical and human error-prone process that requires medical specialists [2]. In addition, the examination of radiology images is a time-limited process that can lead to under-diagnosis in an individual with multiple diseases at the same time.

Recently, with the use of deep learning techniques, researchers have introduced autonomous alternatives to the analysis of images in many medical fields [3]. Promising results have been obtained for deep learning-based disease diagnosis in the fields of classification [4], which focuses only on disease diagnosis and segmentation, which involves the identification of the diseased surface [5,6]. For example, classification, segmentation, and detection-based studies provide only limited information, such as a single word about the presence of the disease or the marking of the diseased surface [7,8,9]. It is far from providing information about the details of the diagnosis. In the field of medical report generation, images are output as a meaningful report consisting of multiple words and sentences [10]. For this reason, the medical report generation workspace is a good decision support system that can be useful for doctors to make decisions about disease diagnosis [11].

In recent years, studies using deep learning architectures, in particular, have achieved successful results in the generation of meaningful text from images [12]. Studies that generate paragraph-level reports from chest X-ray images generally use the encoder–decoder deep learning architecture, which is a two-stage process [13]. The encoder part involves feature extraction from the images. In the decoder part, the text is generated using the features extracted from the texts and the features extracted from the image in the encoder stage [14]. It is very important that the generated reports are regular and meaningful texts and can be understood by humans when they read them. The main tasks of the decoder stage in this field of study are the generation of correct words, the formation of sentences by ordering the generated words in a regular order, the length of the sentences to maintain the integrity of meaning, and the formation of paragraphs by ordering the sentences correctly.

In the encoder stage, researchers have tried to extract the best possible features from images by choosing convolutional neural networks (CNN) or transformer-based architectures. Liu et al. conducted a study that preferred CNN architectures in the encoder part and RNN architecture in the decoder part [15]. Using the Open-I dataset, the researchers obtained a BLEU-1 score of 0.359 in the methods they named NLG and CCR. In another study using the DenseNet101 architecture on the encoder side, the researchers obtained a word overlap evaluation metric BLEU-1 score of 0.347 with the VSGRU models they used on the decoder side [16]. There are also studies that prefer transformer-based architectures instead of CNN architectures at the encoder stage. In the encoder stage, Ucan et al. used the vision transformer architecture, which was first developed in the visual feature extraction of transformer-based architectures [17]. In the decoder stage, the researchers developed a hybrid encoder–decoder architecture by preferring the bard architecture. The developed architectures achieved an evaluation metric score of 0.274 in the ROUGE metric.

Although there are several studies on medical report generation in English from medical images in the current literature, there are no studies on report generation in Turkish. There are limited studies in the field of image captioning, which is a similar field of study. TasvirEt [18] and TRCaptionNet [19] are pioneering studies on Turkish image captioning. However, these studies focus on datasets created with images taken from everyday life such as Flickr8K, MS COCO, and Flickr30K rather than medical images. Textual reporting of images in medical fields is a new and open area for the Turkish language.

In this paper, we propose a hybrid encoder–decoder-based deep learning architecture for generating Turkish medical reports from chest X-ray images. While features are extracted from chest X-ray images in the encoder stage, the decoder stage aims to generate meaningful and fluent Turkish sentences from these features. In this context, Swin Transformer architecture is used in the encoder part and GPT architecture enriched with Turkish language is used in the decoder part. With the developed encoder–decoder architecture, it is aimed to increase the report generation performance. In addition, a chest X-ray dataset with Turkish descriptions was prepared and made available for training and evaluation of the model. The main contributions of this study can be summarized as follows:

(i) Presentation of one of the first comprehensive approaches to generating medical reports in Turkish from chest X-ray images;

(ii) Designing a novel hybrid encoder–decoder architecture that integrates a Swin Transformer-based model as an image encoder and a Turkish GPT-2-based model as a language decoder, and evaluating its performance effects on medical report production in detail;

(iii) Addressing an important gap in the literature by making the dataset created for use in Turkish medical natural language processing available to the open-access research community.

The rest of the paper is organized as follows: Section 2 summarizes the methodologies used in the research. Section 3 presents the results obtained in the experiments and discussion. Section 4 discusses the conclusions and future work.

## 2. Materials and Methods

### 2.1. Turkish Chest X-Ray Images Medical Report Generation Dataset

**Figure 1:**
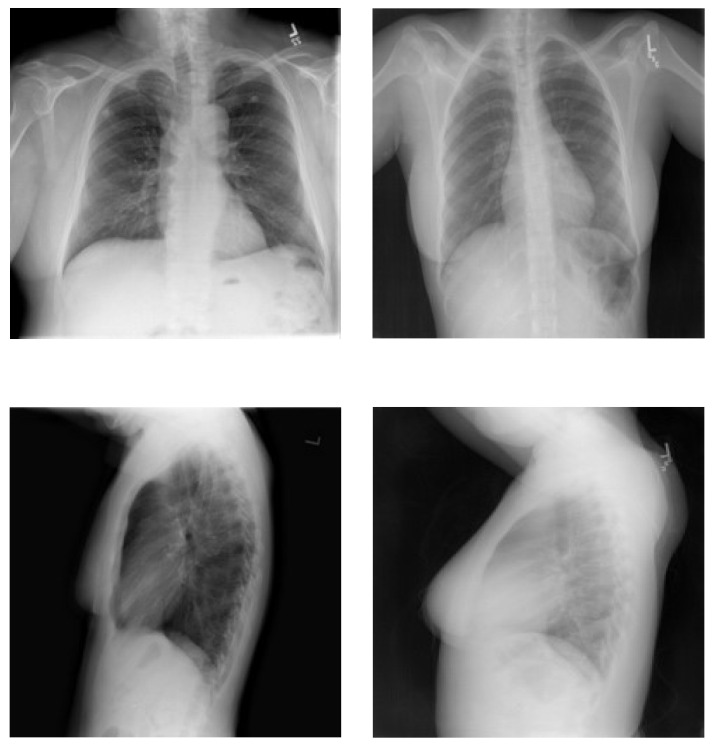
Turkish chest X-ray images reporting dataset randomly selected sample images.

**Figure 2:**
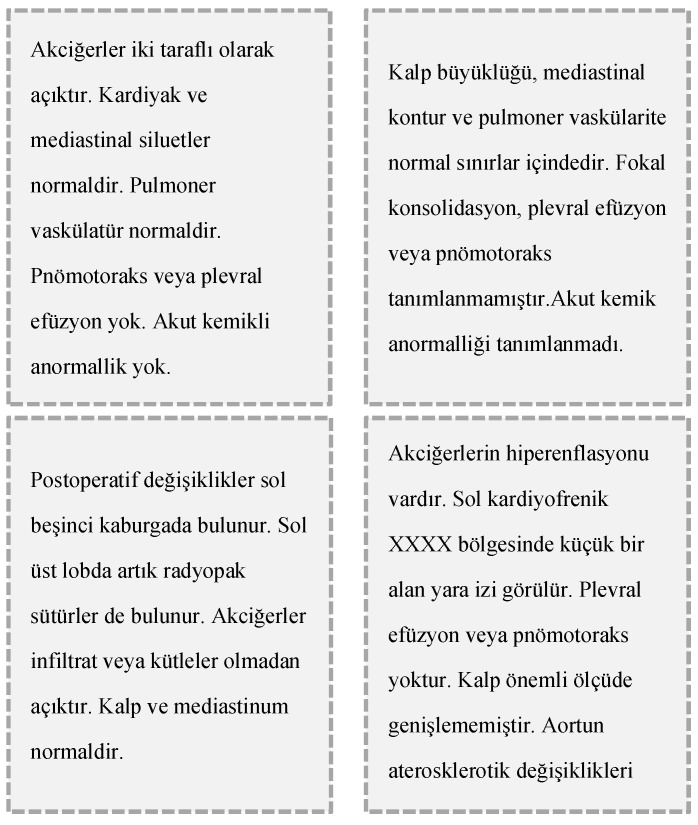
Turkish chest X-ray images reporting dataset randomly selected sample medical reports.

With the ethics committee permission obtained within the scope of our study, medical images of patients and reports related to the images were obtained from Elazığ Fethi Sekin City Hospital. In addition, the image–report pairs in the Indiana University Chest X-Ray dataset [20], which contains chest X-ray images offered as open access in the literature, were also used in the study. All data obtained by the specialist doctor in the field in our research team were checked and approved. Due to the small number of data obtained from the field within the scope of the study, a hybrid data set was created by translating the data in English open-access data sets into Turkish with expert opinion. All chest X-ray images in the dataset were resized to 224 × 224 to create a standard between the images. Textual report data was cleaned from meaningless texts such as introductory phrases, extra spaces, pause expressions, etc., to ensure a standardized structure. The dataset created within the scope of the study contains 5998 image–report pairs in total. Figure 1 below shows the random images in the dataset. Figure 2 shows randomly selected examples of reports written by doctors who are experts in their field.

As part of the study, open-access English image–report matches were translated into Turkish and included in the dataset to address the lack of Turkish chest X-ray report data. This translation process was carried out in multiple stages to ensure both linguistic consistency and medical accuracy. In the first stage, each English report was pre-translated using Google Cloud Translation API, a professional translation tool. These translations were then reviewed in detail by a physician who is a native Turkish speaker and an expert in radiology. The specialist physician, whom we thank in the acknowledgments section of the manuscript, evaluated the medical accuracy of each sentence, corrected any possible meaning shifts, and ensured compliance with Turkish medical reporting terminology. Furthermore, common translation errors identified throughout the process (e.g., ambiguous medical terms, incorrect anatomical expressions) were standardized through a standardized editing process, and the reports were finalized. Thanks to this expert-supported hybrid method, the data generated through translation was structured in accordance with Turkish medical report writing standards, and the reliability of the dataset was ensured.

### 2.2. Swin Transformer

The Swin Transformer architecture, which is effectively used in the field of image processing, was developed by the Microsoft research team [21]. It uses a transformer-based feature extraction mechanism as opposed to convolution operations in traditional CNN-based architectures. The most important point where it differs from other popular transformer-based architectures such as Vision transformer is its shifted window mechanism. In the training phase, the images are processed in progressively smaller claws and features are extracted. One of the most important problems in the analysis of medical images, the problem of identifying fine details while at the same time not losing sight of the overall structure, can be solved thanks to the progressively shrinking window sizes [22]. Swin Transformer architecture was preferred in the encoder part of our encoder–decoder architecture because of its ability to efficiently capture local and global features while reducing computational complexity with the shifted window mechanism in the analysis of medical images.

**Figure 3:**
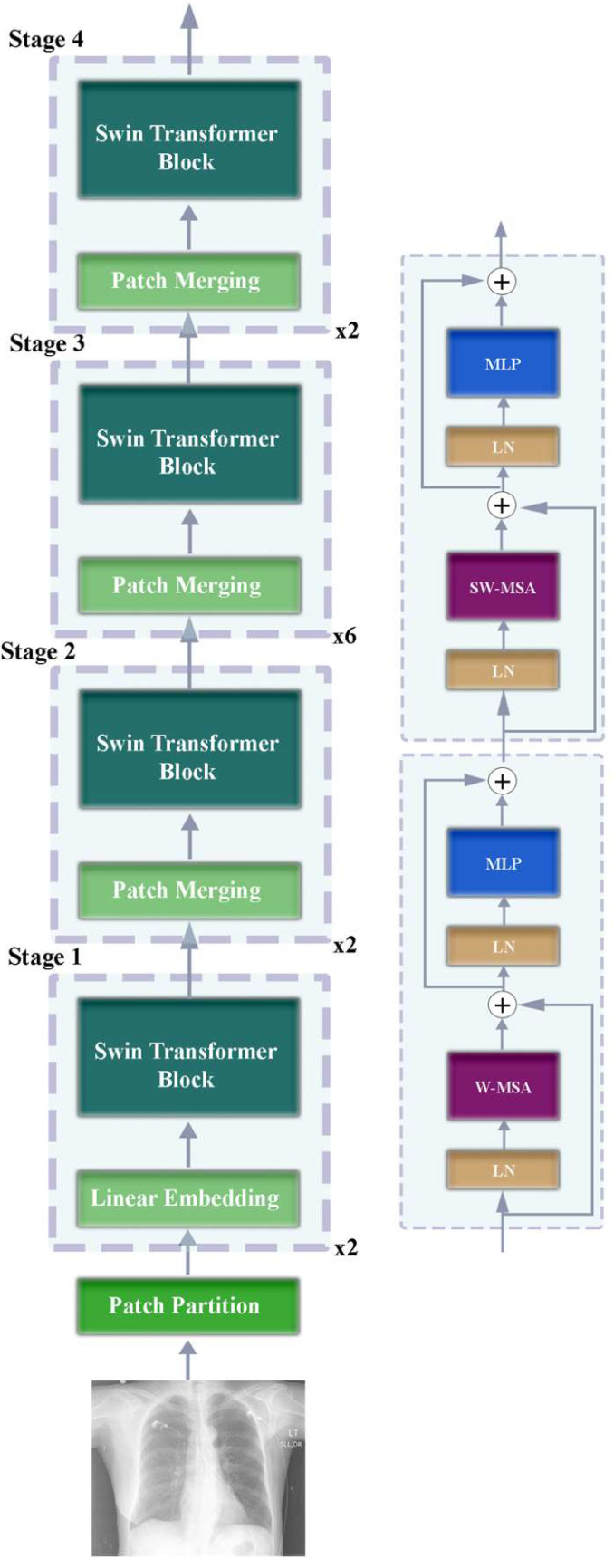
Block diagram of the encoder section of the developed hybrid encoder–decoder model using Swin Transformer architecture.

In the Swin Transformer architecture, the input image is first divided into small non-overlapping patches and transformed into embedding vectors by passing them through a linear layer [23]. These patches are then processed by multiple Swin Transformer layers with hierarchically increasing receptive fields. Each Swin Transformer block contains local window-based self-attention and window shifting operations [24]. After the window shifting step, the architecture continues with a hierarchical fusion step. In the last stage, the obtained features are transmitted to the output layer. This structure contributes to increase the clinical accuracy of reports by better capturing long-range dependencies compared to CNN-based approaches. Figure 3 shows a representative block diagram of the encoder part of the encoder–decoder architecture developed for the Swin Transformer architecture to be used in the Turkish medical reporting task.

### 2.3. Generative Pre-Training Transformer

Generative pre-training transformer (GPT) is an auto-regressive language model developed by OpenAI [25]. The GPT architecture is transformer-based and powered by multilayer attention mechanisms that can easily capture long-range dependencies [26]. The model, which uses a deep neural network structure with a large number of parameters, works with the working logic of predicting the next word correctly based on previous words. Thanks to the advantage of using masked self-attention in the GPT model, each token can only access the tokens before it, preventing information generated during text generation from leaking and corrupting the trained model. The architecture also has the advantage of a multi-head attention mechanism, which allows the learning of language features to proceed in parallel in a multi-head structure, while preserving the sequential structure of the words.

GPT has been successfully used in many deep learning-based natural language processing applications such as text summarization, text generation, and question–answer systems. GPT is trained using large-scale web text sources such as English language books, articles, and web pages, but since GPT architecture is language-agnostic, it can be used in other languages such as Turkish by fine-tuning. In this study, the Swin Transformer encoder and the cosmosGPT model fine-tuned with the Turkish language are used for Turkish medical report generation; cosmosGPT is a GPT model that has been trained and fine-tuned specifically on Turkish texts [27]. Although this model is quite small compared to other large language models, it gives very successful results in Turkish text generation. Various Turkish sources were used to train the model, and care was taken to minimize possible biases. The use of this cosmosGPT model in our study contributed to both computationally efficient and clinically useful results. The features from the image encoder and the language generation capability of cosmosGPT are combined with the hybrid encoder–decoder architecture developed in our study.

## 3. Results and Discussion

In this paper, a hybrid encoder–decoder model is developed to generate Turkish medical reports from chest X-ray images. The encoder part of the proposed model is designed based on Swin Transformer, while the decoder part is based on a fine-tuned Turkish GPT (cosmosGPT) architecture. Our work aims to provide autonomous reporting of diseases that can be detected using chest X-ray images in the Turkish language. Within the scope of our study, a large and comprehensive dataset was obtained by combining medical image–report pairs of patients from Elazığ Fethi Sekin City Hospital located in the Eastern Anatolia region of Turkey and image–report pairs in the Indiana University Chest X-Ray dataset, which is frequently used in the literature. The dataset, which was collected within the scope of the study and planned to be published as open access, was verified by obtaining ethics committee permissions and passing through the controls of the specialist physicians in the field within our research team.

Google Colab Pro+, a paid version that offers high GPU support, was preferred in the training, verification, and testing processes carried out within the scope of the study. NVIDIA A100 GPU and 52 GB RAM were used in all experiments. Equipment such as GPU and RAM were used through a paid membership via a virtual development environment and were not physically sourced. As a result of hyperparameter optimization during model training, the best performance was obtained with batch size 8, learning rate 5 × 10^−5^, optimizer AdamW, weight decay 0.01, and epoch 5. Table 1 shows the optimal hyperparameters used in the proposed architecture.

**Figure 4:**
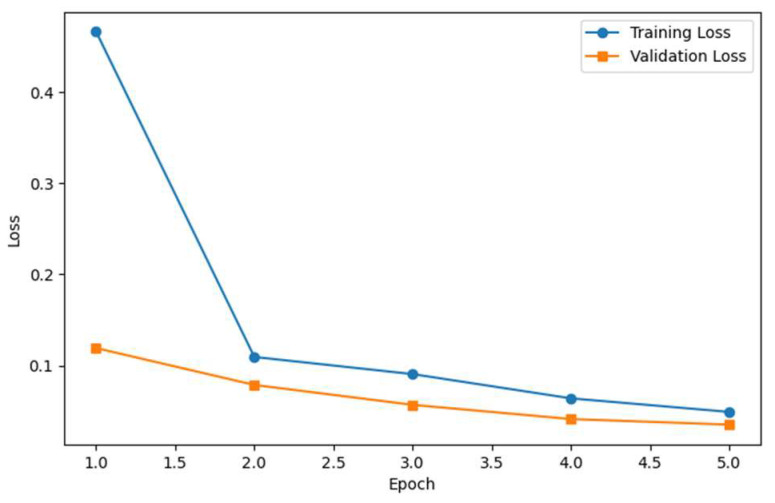
Model-SEY architecture training loss and validation loss graphs.

One of the important indicators of model performance is training and validation loss graphs. By examining training and validation loss graphs, the epoch-based performance of models developed in deep learning architectures can be analyzed. Figure 4 shows the training and validation loss graphs of the Model-SEY architecture developed in our study. The model’s training time took approximately 3 h and 46 min in the experimental environment created with an NVIDIA A100 GPU and 52 GB RAM.

In order to demonstrate the performance of the model developed within the scope of the study with measurable values, word overlap evaluation metrics widely used in the field of natural language processing were utilized. The BLEU metrics used in this context reveal the similarity of the sentences produced by the model with the reference texts at the n-gram level [28]. BLEU-1 uses n-gram weights (1, 0, 0, 0, 0), considering only single words [29]. The BLEU-2 evaluation metric focuses on the relationships between groups of binary words using (0.5, 0.5, 0, 0) n-gram weights. The BLEU-3 metric is based on (0.33, 0.33, 0.33, 0.33, 0) n-gram weights, while BLEU-4 evaluates all four levels equally and uses (0.25, 0.25, 0.25, 0.25, 0.25) weights. The use of BLEU word overlap evaluation metrics allows us to measure the linguistic accuracy of the developed models at the word level and in word groups. The ROUGE metric, with its recall-based approach, measures the level of overlap between autonomous medical report texts and texts written by doctors with word groups [19]. These metrics can be analyzed by researchers to obtain comprehensive and measurable information about the accuracy and adequacy of the model outputs. Table 2 shows the Turkish medical reporting performance of the hybrid encoder–decoder architecture developed within the scope of this study with word overlap evaluation metrics.

Upon analysis of the collected results, it is seen that the developed model performs very well in Turkish medical text generation. The high BLEU-1 score of 0.6412 clearly shows that the model is able to use correct and appropriate words. Although BLEU scores tend to decrease as *n* increases, the BLEU-4 value of 0.3716 shows that the model can produce meaningful outputs even with longer word sequences. The ROUGE score of 0.2240, which is a high value, shows that the model produces outputs that are compatible with the reference texts both at the lexical level and in terms of sentence structure and semantic context. The measurable results obtained show that this model, developed in a field with limited language resources such as Turkish, can make significant contributions to both clinical decision support systems and automatic report generation.

The results obtained with the Model-SEY architecture are the first study in this field in the Turkish language. In order to demonstrate the efficiency of the model with measurable parameters, it was compared with other studies in English found in the literature. Table 3 shares a comparison of the proposed Model-SEY architecture with its major competitors in the field of medical report generation. Table 3 also allows for a comparison of the approaches used in the encoder and decoder sections of the existing models in the literature and the proposed model.

The Model-SEY architecture is optimized for Turkish language report generation by using Swin Transformer on the encoder side and cosmosGPT on the decoder side. When evaluated in terms of BLEU and ROUGE scores, our proposed Model-SEY architecture is particularly effective in BLEU-3 (0.4395) and BLEU-4 (0.3716) scores. It also performs at similar or higher levels than its strong competitors in the literature in terms of BLEU-1 and BLEU-2 scores. However, the relatively low ROUGE score can be attributed to the uniqueness of the Turkish language structure and the differences in preprocessing rules.

In addition, our model stands out not only in terms of success metrics but also in terms of architectural innovation. The Swin Transformer-based encoder structure provides more localized attention regions in the image space. The cosmosGPT-based decoder has the capacity to produce more fluent and contextually consistent sentences. The combined use of these components has increased the model’s ability to produce long and detailed Turkish reports.

The first models in the table use CNN-RNN and TieNet CNN-based encoders and classic sequential processors such as RNN or LSTM. These models show limited success in report generation with low BLEU-3 and BLEU-4 scores. This can be explained by the weakness of classic RNN-based decoders in generating long and context-sensitive medical sentences. Architectures such as VSGRU, CDGPT2, Gamma Enhancement, Vi-Ba, and CNN/RNN with Greedy Search have improved feature extraction success from images with more powerful encoder (DenseNet, Vision Transformer) and transformer-based decoder (GPT, BERT, BART) structures. In particular, the Gamma Enhancement model shows remarkable performance, reaching a BLEU-4 score of 0.412. However, ROUGE scores have either not been reported or have been limited in most models. This indicates that production at the meaning level has not been sufficiently measured, rather than word overlap. Current models such as G-CNX achieve powerful results using a ConvNeXt encoder and a GRU decoder with deeper and more evolved architectural designs. However, the success of this model is specific to the English language, and its generalizability to languages with different structures, such as Turkish, is uncertain.

Model-SEY, on the other hand, proposes a structure optimized for Turkish report generation for the first time in this field, using the Swin Transformer architecture in the encoder part and the cosmosGPT large language model in the decoder part. High scores were obtained in all metrics from BLEU-1 to BLEU-4, with BLEU-2 and BLEU-3 scores being higher than many English models. This demonstrates the model’s success in both extracting meaningful visual features from images and converting these features into Turkish natural language output.

**Figure 5:**
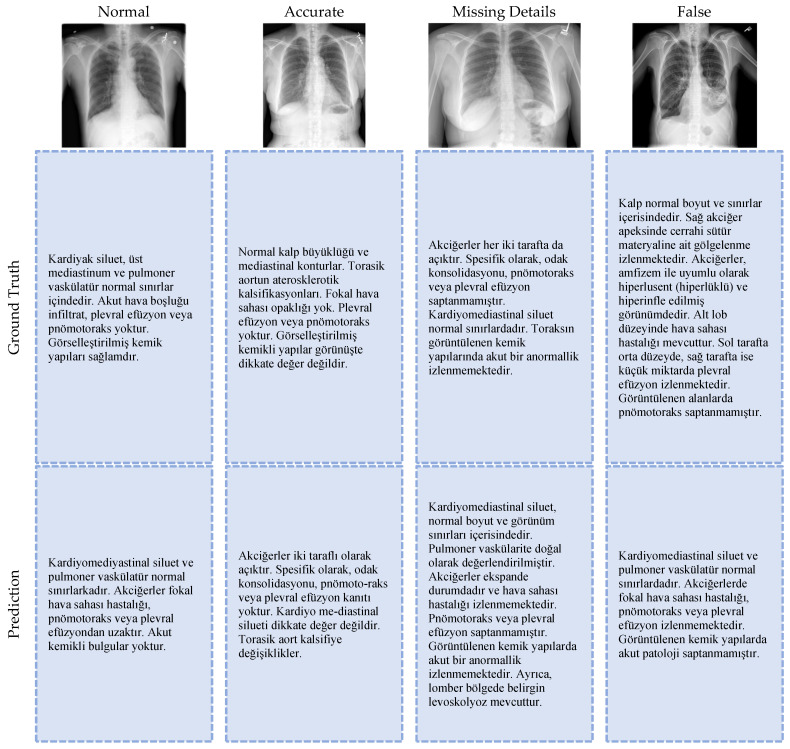
Sample medical reports generated by Model-SEY.

In addition to the measurable parameters, the outputs were also checked by observational analysis of the expert doctors in our study team and it was confirmed that medical reports were generated with a success that could support doctors in decision making during the diagnosis phase. Figure 5 shows four randomly selected sample images, reports written by doctors, and medical reports generated by the proposed architecture.

To assess the clinical accuracy of the model outputs, a qualitative analysis was conducted in addition to automatic evaluation metrics like BLEU and ROUGE, which are only based on textual similarity. In this sense, professional radiologists clinically assessed reports produced by Model-SEY for a chosen subset of chest X-rays. Based on the image’s findings, each report was categorized into four groups: normal, accurate, missing details, and false. These groups were determined by whether or not the reports contained accurate and comprehensive information. These categories seek to assess the model outputs’ clinical reliability, applicability, and linguistic sufficiency. Selected examples from this evaluation process are shown in Figure 5. The original chest X-ray, the reference report, the Model-SEY report, and the expert evaluation category are all included in each example. This analysis shows that the model can produce reliable and clinically appropriate reports in many situations, but it also shows that in certain instances, there may be limited detail deficiencies or incorrect interpretations. Therefore, beyond textual similarity scores, this expert validation offers a more thorough and clinically focused evaluation of model performance.

The clinical impact of this study is to reduce the time and workload of specialized physicians when interpreting chest X-ray images. It will also provide decision-making support in the diagnostic phase, preventing the underdiagnosis or misdiagnosis of diseases. In addition, the proposed architecture accelerates the diagnostic process in regions where healthcare personnel are limited and will contribute to the prevention of possible human errors. In future work, the aim is to integrate this architecture not only into chest X-rays but also into other types of medical imaging such as brain MRI, CT, and mammography. It will also shed light on future work in the field of Turkish medical image captioning and autonomous medical report generation.

## 4. Conclusions

This study proposes a deep learning architecture framework that produces autonomous reports in Turkish by training image–report pairs. The encoder-–decoder-based architecture, named Model-SEY, was developed within the scope of this study. The Swin Transformer architecture was used in the encoder section, while the cosmosGPT architecture, a Turkish architecture, was used in the decoder section. As a result of the training and testing process, a BLEU-1 word overlap evaluation metric score of 0.6412 was obtained. The experimental results show that the proposed model can support doctors in their decision-making processes in the diagnosis and reporting of diseases.

The first limitation of the study is that the model was trained and tested using only transformer-based deep learning architectures. The success of report generation in traditional CNN-based architectures is unknown. In addition, the system was run in a laboratory environment using image–report pairs obtained from hospitals in advance. There is limited information on real-time training and testing processes. Future studies plan to conduct a new study on the detection and reporting of tumorous regions in brain MRI images.

## Figures and Tables

**Table 1:** Hyperparameter configuration for Model-SEY.

Hyperparameter	Configuration
Learning rate (LR)	5 × 10^−5^
Optimizer	AdamW
Weight decay	0.01
Number of epochs	5
Activation function	GELU
Batch size	8

**Table 2:** Word overlap evaluation metric results of Turkish medical generation hybrid encoder–decoder architecture.

Word Overlap Evaluation Metric	Value	Value (%)
BLEU-1	0.6412	64.12
BLEU-2	0.5335	53.35
BLEU-3	0.4395	43.95
BLEU-4	0.3716	37.16
ROUGE	0.2240	22.40

**Table 3:** Comparison of architectural details and reporting achievements of previous projects with our Model-SEY architecture.

Method	Year	Target Language	Encoder	Decoder	BLEU-1	BLEU-2	BLEU-3	BLEU-4	ROUGE
CNN-RNN [30]	2015	English	CNN	RNN	0.316	0.211	0.140	0.095	0.267
VSGRU [16]	2021	English	DenseNet	GRU	0.347	0.221	0.156	0.116	0.251
CDGPT2 [16]	2021	English	DenseNet	GPT	0.387	0.245	0.166	0.111	0.289
TieNet [14]	2018	English	CNN	LSTM	0.286	0.159	0.103	0.073	0.226
Gamma Enhancement [2]	2024	English	DenseNet	BERT	0.363	0.371	0.388	0.412	-
Vi-Ba [17]	2024	English	Vision Transformer	BART	-	-	-	0.150	0.274
CNN/RNN and Greedy Search [1]	2023	English	CNN	RNN	0.592	0.422	0.298	0.205	-
G-CNX [10]	2025	English	ConvNeXt	GRU	0.6544	0.5035	0.3682	0.2766	0.4277
(Our) Model-SEY	2025	Turkish	Swin Transformer	CosmosGPT	0.6412	0.5335	0.4395	0.3716	0.2240

## Data Availability

The data presented in this study are available on request from the corresponding author, due to the fact that this is a new area of research and a very current topic.
